# Liquid Phase Isolation of SnS Monolayers with Enhanced Optoelectronic Properties

**DOI:** 10.1002/advs.202201842

**Published:** 2022-12-27

**Authors:** Abdus Salam Sarkar, Ioannis Konidakis, E. Gagaoudakis, G. M. Maragkakis, S. Psilodimitrakopoulos, D. Katerinopoulou, L. Sygellou, G. Deligeorgis, Vassilios Binas, Ilias M. Oikonomou, Philomela Komninou, G. Kiriakidis, G. Kioseoglou, E. Stratakis

**Affiliations:** ^1^ Institute of Electronic Structure and Laser Foundation for Research and Technology‐Hellas Heraklion 700 13 Greece; ^2^ Department of Physics University of Crete Heraklion 710 03 Greece; ^3^ Institute of Chemical Engineering Sciences (ICE‐HT) Foundation of Research and Technology Hellas, P.O. Box 1414 Rio Patras 26504 Greece; ^4^ Department of Physics Aristotle University of Thessaloniki Thessaloniki 54124 Greece; ^5^ Department of Materials Science and Technology University of Crete Heraklion 710 03 Greece

**Keywords:** anisotropic 2D materials, carrier transport, field effect transistor (FET), microscopy, monolayer SnS photodetectors

## Abstract

Recent advances in atomically thin two dimensional (2D) anisotropic group IV_A_‐VI metal monochalcogenides (MMCs) and their fascinating intrinsic properties and potential applications are hampered due to an ongoing challenge of monolayer isolation. Among the most promising MMCs, tin (II) sulfide (SnS) is an earth‐abundant layered material with tunable bandgap and anisotropic physical properties, which render it extraordinary for electronics and optoelectronics. To date, however, the successful isolation of atomically thin SnS single layers at large quantities has been challenging due to the presence of strong interlayer interactions, attributed to the lone‐pair electrons of sulfur. Here, a novel liquid phase exfoliation approach is reported, which enables the overcome of such strong interlayer binding energy. Specifically, it demonstrates that the synergistic action of external thermal energy with the ultrasound energy‐induced hydrodynamic force in solution gives rise to the systematic isolation of highly crystalline SnS monolayers (1L‐SnS). It is shown that the exfoliated 1L‐SnS crystals exhibit high carrier mobility and deep‐UV spectral photodetection, featuring a fast carrier response time of 400 ms. At the same time, monolayer‐based SnS transistor devices fabricated from solution present a high on/off ratio, complemented with a responsivity of 6.7 × 10^−3^ A W^−1^ and remarkable stability upon prolonged operation in ambient conditions. This study opens a new avenue for large‐scale isolation of highly crystalline SnS and other MMC manolayers for a wide range of applications, including extended area nanoelectronic devices, printed from solution.

## Graphical abstract

A novel liquid phase exfoliation approach that enables overcoming the strong interlayer binding energy among the SnS layers. The successful isolation of high crystalline atomically thin SnS single layers at large quantities open a new avenue for large‐scale production of other MMC nanolayers for a wide range of applications, including extended area of nanoelectronic devices, printed from solution.

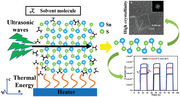

## 1 Introduction

Since the successful isolation of graphene, the isolation of beyond‐graphene materials, such as boron nitride (BN), MMCs, transition metal dichalcogenides (TMDs), transition metal trichalcogenides, ternary bismuth telluride halides, and phosphorene, has opened up a new horizon in 2D materials research.^[^ ^1^, ^2^, ^3^, ^4^ ^]^ Among them, the isolation of single layers of anisotropic 2D materials has been a major challenge, providing unique opportunities for fundamental physical insights and novel nanoelectronic device applications.^[^ ^5^, ^6^, ^7^, ^8^, ^9^, ^10^ ^]^ In particular, the MMCs with chemical formula MX (M = Ge, Sn, and so on, and X = S, Se) have gained considerable attention due to their peculiar intrinsic physical properties at the single atomic layer.^[^ ^6^, ^8^, ^11^, ^12^, ^13^ ^]^ The exceptional physical responses originate from the in‐plane structural anisotropy (Figure S1, Supporting Information) exhibited by such 2D crystals, which is rooted in the puckered lattice structure. Theoretical investigations reveal several unusual intrinsic physical properties at their quantum limit,^[^ ^6^, ^11^, ^13^, ^14^, ^15^, ^16^, ^17^, ^18^, ^19^ ^]^ which have not been proved by experiments yet. This can be attributed to the limited success in the isolation of atomically thin layers, due to the strong interlayer interactions present in such materials.

Among prominent MMCs, the 2D black phosphorus (BP) analogous, SnS,^[^ ^11^ ^]^ has received significant attention for the development of anisotropic photonic and optoelectronic devices.^[^ ^8^, ^10^, ^20^, ^21^ ^]^ SnS consists of two elements with different electronegativity, as a result of the broken inversion symmetry in odd layers ($D_{2\upsilon }^{7}$), a feature that gives rise to rich and intriguing physical phenomena, including room temperature spin valley polarization (bulk),^[^ ^22^ ^]^ dichroism,^[^ ^23^ ^]^ ferroelectricity,^[^ ^20^, ^24^ ^]^ ferroelasticity,^[^ ^25^ ^]^ saturable absorption,^[^ ^26^ ^]^ and piezophototronics.^[^ ^27^, ^28^ ^]^ Besides this, the orthorhombic crystal structure (Pnma) with low crystal symmetry C_2*υ*_ in this material, enables the exploration of the new order parameter of spin‐orbital coupling and polarizability.^[^ ^11^ ^]^ Furthermore, being a p‐type semiconductor, 1L‐SnS shows both direct and indirect bandgap properties^[^ ^29^ ^]^ with high absorption coefficient^[^ ^30^ ^]^ and band tunability,^[^ ^31^ ^]^ which makes it ideal for photodetectors^[^ ^10^, ^32^ ^]^ and photovoltaic cells.^[^ ^33^ ^]^ At the same time, the phonon‐limited carrier mobility in a monolayer SnS is in the order of 10^3^–10^5^ cm^2^ V s^−1^,^[^ ^17^ ^]^ which is significantly higher than most 2D materials. All the above prominent properties render 1L‐SnS to be a promising candidate for unique optoelectronic applications.

To date, several synthesis methodologies have been applied to realize 1L‐SnS. In particular, chemical vapor deposition (CVD)^[^ ^20^ ^]^ and atomic layer deposition (ALD)^[^ ^34^ ^]^ have failed to grow 1L‐SnS, due to the uncontrolled growth along the armchair and zig‐zag crystallographic directions. Although physical vapor deposition (PVD) has been reported to grow 1L‐SnS,^[^ ^21^ ^]^ the crystalline quality is poor, while the extended production of sheets on arbitrary substrate is not yet feasible. At the same time, the strong interlayer binding energy in MXs and thus SnS,^[^ ^35^ ^]^ makes the scotch‐tape mediated micromechanical exfoliation of 1L‐SnS almost impossible.^[^ ^22^, ^23^, ^27^, ^36^ ^]^ Liquid metal‐based synthesis (LMS) presented a promising approach for growing large‐area ultrathin layers of SnS.^[^ ^32^, ^37^ ^]^ However, the significantly high growth temperatures (on the order of 350 °C), coupled with an extremely inert environment, required by LMS, increase the production cost and are not compatible with flexible electronics adopting thermally fragile substrates.^[^ ^38^ ^]^ In contrast, liquid phase exfoliation (LPE) has presented an excellent approach for isolating large quantities of monolayer 2D materials from their bulk counterparts.^[^ ^39^, ^40^, ^41^, ^42^, ^43^ ^]^ Using LPE, the exfoliation of ultrathin, bi‐ and multi‐ layers of SnS has been recently reported.^[^ ^26^, ^44^, ^45^ ^]^ Nevertheless, the successful LPE isolation of high‐quality, atomically thin 1L‐SnS and the subsequent study of its intrinsic electronic and optical properties, coupled with device performance, still remain a challenge.

In addition, in recent years, the potential of layered materials is revived for the next generation consumer electronic applications, placing particular emphasis on flexible and wearable devices.^[^ ^39^, ^46^, ^47^ ^]^ In this context, novel layered materials inks can be an ideal route for industrial‐scale production of such devices, while the LPE technique represents the ideal method for large‐scale commercial electronic applications.^[^ ^48^, ^49^ ^]^ More important, isolated 2D materials inks can be deposited on both rigid and flexible substrates via the spin or spray coating methods that be easily scalable and industrially compatible processes. Another matter, from the scale‐up point of view, is the choice of the ink solvent, as the solvent boiling point critically affects the device fabrication process. In particular, the commonly used high boiling point solvents such as N‐methyl‐2‐pyrrolidone, dimethylsulfoxide, and dimethylformamide leave contaminants on the surface of deposited 2D flakes, which adversely affect the device performance. Hence, the isolation of novel 2D inks in low boiling point solvents is desirable.

In this communication, we present a new LPE methodology for the successful isolation of single‐unit cell 1L‐SnS, with lateral dimensions of a few hundred nanometers. It is based on the application of external thermal energy in synergy with the hydrodynamic force applied due to and during ultrasonication. We observe that the optimal combination of those two energy sources is sufficient to overcome the strong interlayer binding energy of SnS. The intrinsic physicochemical properties and crystal structure of 1L‐SnS, produced by such a thermally assisted LPE (T‐LPE) method, have been systematically explored via a series of microscopy and spectroscopy techniques, while the optoelectronic performance has been investigated through the fabrication and evaluation of field effect transistor (FET) and photodetector devices. Based on the analytical studies performed, the in‐plane anisotropic nature of the 1L‐SnS crystal properties along armchair and zig‐zag directions have been investigated and discussed. It is demonstrated that the low‐cost and controllable monolayer isolation, coupled with the appreciable optoelectronic performance and anisotropic properties, makes 1L‐SnS, prepared by T‐LPE, a promising 2D material for future solution‐processed, low‐cost, and nanoelectronic applications.

## 2 Results and Discussion

Most recently, the isolation of a monolayer MX has proven to be difficult due to the strong interlayer coupling attributed to the remarkable difference in electronegativity between the two atoms M and X.^[^ ^8^, ^36^ ^]^ For SnS in particular, the chalcogen atom, S, features a much stronger electronegativity than Sn, as a result of the fact that S (4d^10^5s^2^5p^4^) captures two electrons from Sn (4d^10^5s^2^5p^2^) and leads to a drastic change in its electronic configuration (4d^10^5s^2^5p^0^). A summary of the various techniques employed to isolate SnS thin layers and the corresponding limitations and properties are summarized in Tables S1 and S2, Supporting Information. In this study, the isolation of 1L‐SnS is explored by a solvent‐based LPE approach, which was stimulated with external thermal energy during cavitation. In particular, SnS powder was initially dissolved in acetone, which is a suitable solvent to prevent restacking and aggregation, due to its surface tension and Hansen solubility parameters being compatible with SnS.^[^ ^42^ ^]^ Subsequently, SnS dispersions were ultra‐sonicated at a bath temperature of 50 °C, to create cavitation events for 10 or 20 h. Following the sonication process, a dark brown solution was obtained, which was centrifuged at an optimized speed of 8000 rpm for 15 min. The results were compared with dispersions obtained at the same conditions, however at a bath temperature below 25 °C. The effectiveness of the exfoliation process and the quality of the isolated ultrathin SnS layers were evaluated by morphological, chemical, and structural analyses.

**Figure 1:**
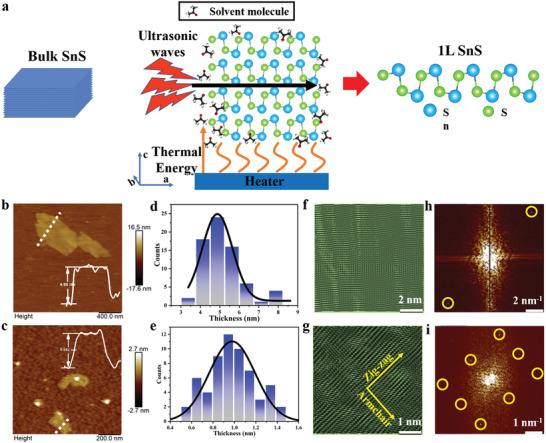
a) Schematic representation of the thermally‐assisted LPE isolation of monolayer SnS. Blue and green circles represent the tin and sulfur atoms respectively; Representative AFM images of SnS nanosheets obtained using b) 10 and c) 20 h cavitation times, both centrifuged at 8000 rpm. The inset shows the height profile (white solid line) along the section indicated by the white dashed line; Histograms of the thickness distribution of SnS nanosheets obtained using 10 h (average thickness ≈4.830 ± 0.058 nm), d) and 20 h (average thickness ≈0.904 ± 0.014 nm), e). Solid curves represent Gaussian fittings to the data. The corresponding lateral dimensions are 1.093 ± 0.108 µm and 238 ± 86 nm for 10 h‐ and 20 h‐ cavitated samples (Figure S2, Supporting Information); Lattice‐resolution AFM images obtained from selected areas in b) (f) and c) (g), respectively; Fast Fourier transform of the lattice resolution images shown in f) and g) for FL‐ (h) and 1L‐, (i) SnS respectively.

Atomic force microscopy (AFM) imaging of the exfoliated SnS flakes and related statistical analysis showed that a crystal thickness control, from few to single layers, could be realized upon controlling the cavitation and centrifugation times. **Figure** **1**b,c show representative AFM topography images of SnS flakes produced using 10 and 20 h cavitation times, at 8000 rpm centrifugation speed, respectively. The corresponding distributions of the measured thicknesses values are shown in Figure 1d,e. It can be observed that the average sheet thickness is reduced from a few nm to that of a single layer (< 1 nm) upon increasing the cavitation time. Further analysis showed that the average thickness of the isolated SnS nanosheets is decreasing upon increasing the centrifugation speed (Figure S2, Supporting Information). In particular, the average sheet thickness obtained using 20 h of cavitation time and 8000 rpm centrifugation speed was measured to be ≈0.904 ± 0.014 nm. An orthorhombic single‐unit cell of 1L‐SnS comprises two layers of SnS stacked one on top of the other (Figure S1c, Supporting Information), containing four tin (Sn) and sulfur (S) atoms, independent of their stacking sequence. Thus, the above sheet thickness corresponds approximately to that of a unit cell of 1L‐SnS (i.e., 2 × 0.260 + 0.276 nm) plus the van der Waals gap with the substrate of ≈0.2 nm (Figure S1c, Supporting Information).^[^ ^21^, ^24^, ^32^, ^37^ ^]^ By contrast, the SnS nanosheets prepared at the lower cavitation time exhibited an average thickness of ≈4.830 ± 0.058 nm, corresponding to multiple unit cells within few‐layered SnS (FL‐SnS). At the same time, the lateral dimensions of the isolated flakes are increased with the decrease of the cavitation time, from a few hundred nanometers to close to a micrometer for 1L‐SnS and FL‐SnS, respectively (Figure S3, Supporting Information).

It is found that the average thickness of the exfoliated SnS flakes is strongly affected by the external thermal energy applied during the cavitation process. In particular, the SnS sheets isolated using 20 h of cavitation time and centrifugation speed of 8000 rpm, but at lower bath temperature (< 25 °C), presented an average thickness of 3.88 ± 0.69 nm, corresponding to FL‐SnS crystals (Figure S4a,b, Supporting Information). At the same time, the average lateral dimensions of the flakes obtained exceeded half of a micron. To understand the underlying exfoliation mechanism of SnS, we looked at the recent advances in LPE of such materials, summarized in Table S4, Supporting Information. It has been well established that the cavitation time played a significant role in the efficient LPE of 2D materials and MXs in particular. However, none of the LPE approaches to date could give rise to the successful LPE isolation of high‐quality and volume MXs monolayers. This is due to the strong interlayer binding energy of such materials (Figure S1a, Supporting Information), attributed to the presence of two elements of different electronegativity within the crystal structure. It is found that the calculated IBE of SnS is much higher than the graphene and beyond graphene 2D TMDs.^[^ ^50^, ^51^ ^]^ Besides this, the bath sonication temperature has been also shown to play a significant role in the efficiency of the LPE process in graphene^[^ ^52^, ^53^ ^]^ and other 2D materials. To date, however, there is no such study for the LPE of MXs. Indeed, it has been demonstrated that the bath temperature provides an additional driving force acting in synergy with the cavitation one.^[^ ^52^ ^]^ SnS crystal, in particular, features a significantly high thermal expansion coefficient along its adjacent layers.^[^ ^54^ ^]^ As a result, the first‐order temperature coefficient, which is directly related to the lattice thermal expansion is significantly higher in SnS than in the other 2D materials.^[^ ^26^ ^]^ This superior physical property of SnS can act as an additional perturbation force during the LPE, via the thermodynamic expansion of the homointerface among the 2D crystal planes. Therefore, the synergetic effect of the remarkable thermal expansion of the crystal planes and the hydrodynamic force proved to be sufficient to overcome the strong interlayer binding energy among the SnS bilayer crystal planes. A pictorial representation of the proposed mechanism for the exfoliation process is shown in Figure 1a. It should be noted here that the MXs crystals commonly contain planes of atoms along each layer, bonded with a weaker than the intra‐layer atomic force.^[^ ^35^, ^55^ ^]^ This could explain the decrease in the lateral dimensions of the exfoliated nanosheets as the cavitation time is increased, for a specific bath temperature. The thermally assisted LPE technique, introduced here, may be useful to isolate a single layer of other high‐binding‐energy 2D materials, including MXs.

**Figure 2:**
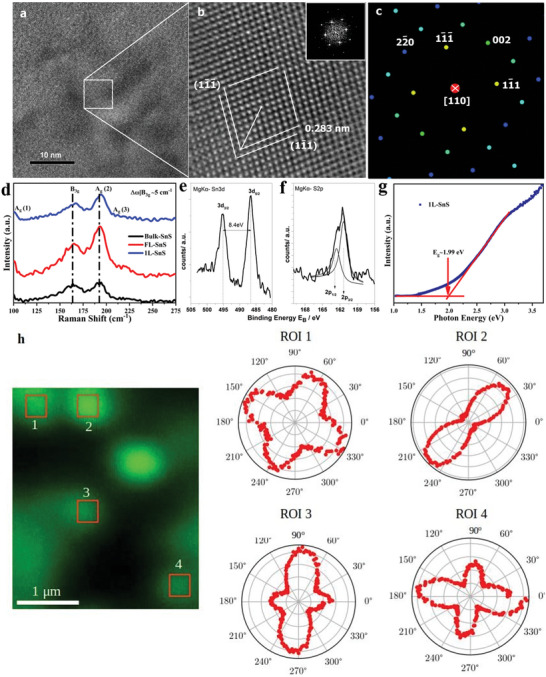
a) HRTEM image illustrating the atomic structure of an isolated SnS nanosheet projected along the [110] zone axis; b) Magnified image of the white square frame depicted in a). The spacing of the two^[^ ^57^ ^]^ type lattice fringes and the armchair direction of the orthorhombic structure are indicated. The corresponding FFT is shown as inset; c) Simulated selected area electron diffraction (SAED) pattern of the [110] zone axis oriented with respect to the HRTEM image; d) Raman spectra of bulk, isolated FL‐SnS and 1L‐SnS. The three optically active Raman vibrational peaks correspond to B_3g_, A_g_ (1), and A_g_ (2) modes; e) XPS spectra of SnS nanosheets, showing the core level Sn‐3d_3/2_ and Sn‐3d_5/2_ peaks; f) XPS spectrum of the core level S‐2p; g) Tauc plot of 1L‐SnS calculated from UV–vis absorption spectra. The estimated optical band gap is ≈1.99 eV; h) (left) Experimental SHG image of 1L‐SnS crystals belonging in the same field of view. Brighter color indicates higher SHG intensity. (Right) Experimental data (in red dots) of the P‐SHG intensity taken from each ROI depicted in the image, presented in polar plots as a function of the angle of excitation linear polarization. Interestingly, the shape of the polar diagrams changes for different flakes, which is the signature of their in‐plane anisotropy.

The puckered atomic structure of ultrathin SnS flakes has been investigated using high‐resolution transmission electron microscopy (HRTEM), image processing, and image simulations. An HRTEM image, recorded from an isolated single crystalline SnS nanoflake recorded along the [110] zone axis (z. a.) of the Pnma orthorhombic structure, is illustrated in **Figure** **2**a. In this structure, the angle between the [110] viewing direction and the [100] normal to the flake is equal to 19.68°. Magnification of the white square frame of Figure 2a is presented in Figure 2b revealing the projected atomic structure of the nanoflake. The (002), (2$\bar{2}$0), (1$\bar{1}$1), and (1$\bar{1}\bar{1}$) lattice fringes are clearly resolved and the spacing of the corresponding lattice planes was determined as 0.212, 0.191 and 0.283 nm (indicated by white lines) respectively.^[^ ^56^ ^]^ The white arrow designates the [001] armchair direction of the structure. The inset FFT illustrates the spatial frequencies that compose the HRTEM image. It reveals the single crystalline nature of the nanoflake orthorhombic atomic arrangement. The angle between the (1$\bar{1}$1) and (1$\bar{1}\bar{1}$) lattice fringes was measured equal to 84°, between the (002) and (1$\bar{1}$1) equal to 48°, and between (2$\bar{2}$0) and (1$\bar{1}\bar{1}$) equal to 42°, which agreed well to the theoretical Pnma model as confirmed by the corresponding simulated selected area electron diffraction (SAED) pattern shown in Figure 2c.

Further confirmation of the observed SnS nanoflakes structure and thickness is given in Figure S1, Supporting Information, where the balls and sticks supercell model of the Pnma SnS structure, projected along the [110] zone axis, is presented (Figure S1b, Supporting Information). This model was used for the simulation of the HRTEM image and its correlation to the experimental one. The simulated image of the projected potential of the supercell (the electrostatic potential of a crystal) is shown in Figure S1d, Supporting Information, while the experimental HRTEM image with superimposed inset of the corresponding simulated, for a nanoflake thickness of 1.2 nm (2 MLs), are illustrated in Figure S1e, Supporting Information.

Lattice‐resolution AFM images of the isolated flakes were additionally obtained, as shown in Figure 1f,g, for 10h‐ and 20h‐ cavitated samples respectively. The corresponding FFT of the image corresponding to 1L‐SnS (Figure 1i) reveals a highly crystalline orthorhombic structure, in agreement with the HRTEM analysis presented above. Indeed, the corner angle between the armchair and zig‐zag directions was measured to be ≈87°, thus identical to the value obtained by HRTEM analysis. Notably, the lattice resolution images obtained from different locations within the same sheet of 1L‐SnS were found to exhibit identical crystallographic features. The above observations unambiguously confirmed the consistency in identifying the crystal structure of 1L‐SnS, via the use of lattice resolution AFM images. It should be emphasized that the orthorhombic crystal structure cannot be revealed by the FFT analysis of the lattice resolution AFM images obtained from the thicker SnS flakes prepared (Figure 1h). Therefore, the identification of the crystal structure, via lattice resolution AFM topography images, is limited to 1L‐SnS nanosheets.

Raman spectroscopy has been extensively employed to confirm the successful isolation of layered 2D materials.^[^ ^58^, ^59^, ^60^ *^]^* In particular, SnS belongs to the orthorhombic crystal structure *Pnma* with low crystal symmetry (C_2*υ*_) showing 24 phonon modes at the center of the Brillouin zone (BZ), Γ, expressed as^[^ ^58^ ^]^

(1) $$\Gamma =4A_{g}+2B_{1g}+4B_{2g}+2B_{3g}+2A_{u}+4B_{1u}+2B_{2u}+4B_{3u}$$

where *A_g_*, *B*_1*g*_, *B*_2*g*_, and *B*_3*g*_ are the optically active Raman modes. The room temperature Raman spectra of isolated SnS sheets are presented in Figure 2d, where three sharp optically active phonon modes are clearly resolved for bulk, FL‐ and 1L‐SnS nanosheets. In particular, the phonon modes peaked at ≈100 and ≈194, which correspond to A_g_ (1) and A_g_ (2) vibrational modes, respectively, while the peak at ≈167 cm^−1^ is associated with the B_3g_ orthorhombic one.^[^ ^20^, ^37^, ^61^ ^]^ In the case of bulk SnS the peaks that appeared at 162 and 192 cm^−1^ correspond to B_3g_ and A_g_ phonon modes, respectively. Due to the detection limit of the spectrometer used, the A_g_ (1) mode at ≈100 cm^−1^ cannot be monitored in this case, as it is shifted to lower wavenumbers. Besides this, the B_3g_ mode is observed to be blue‐shifted by ≈3 and ≈5 cm^−1^, with respect to that of the bulk, for the isolated FL‐ and 1L‐ SnS sheets, respectively. The same occurs for the A_g_ (2) mode, which is blue‐shifted by ≈1 and ≈2 cm^−1^ respectively. Such blue shifts can be attributed to the reduction of the dielectric screening effect upon decreasing the crystal thickness, which is consistent with the thickness lowering of the exfoliated crystals.^[^ ^58^, ^62^ ^]^ Finally, it was observed that the relative intensity of the Raman peaks changes when sheets are thinned down from bulk to monolayer. In particular, as shown in Figure S5, Supporting Information, the B_3g_/A_g_ (2) peak intensity ratio is enhanced upon crystal thinning, possibly attributed to respective changes in the interlayer coupling.

In order to shed light on the anisotropic nature of a buckled layered structure,^[^ ^10^, ^20^, ^21^, ^22^, ^56^, ^63^ ^]^ it is necessary to identify the difference in the in‐plane structural properties between the armchair and zig‐zag crystal lattice directions. Although the angle‐resolved polarized Raman^[^ ^21^, ^58^, ^64^ ^]^ as well as polarization‐resolved absorption and photoluminescence spectroscopies can determine such differences in the crystal symmetry,^[^ ^65^, ^66^ ^]^ the chemically exfoliated 2D sheets of this study are much smaller in lateral dimensions than the diffraction‐limited beam spot size of ≈1 µm. As an alternative, here the in‐plane structural anisotropy of the exfoliated crystals is explored by polarized second harmonic generation (P‐SHG).^[^ ^67^ ^]^ In Figure 2h, we present the sum of the P‐SHG intensity images, obtained from different SnS crystals belonging to the same field of view, for all orientations of the excitation linear polarization *φ*. It is observed that the shape of the polar‐diagrams changes for different nanosheets, which is the signature of differences in their in‐plane anisotropy.^[^ ^67^ ^]^

X‐ray photoelectron spectroscopy (XPS) measurements were carried out to investigate the chemical states and stoichiometry of the isolated SnS nanosheets. Figure S6, Supporting Information presents the Sn3d and S2p core level spectra of the isolated FL‐SnS sheets, confirming the high purity of isolated SnS crystals. The core level Sn‐3d spectrum of 1L‐SnS (Figure 2e) depicted two peaks at the binding energies of 486.5 and 494.9 eV, corresponding to Sn‐3d_5/2_ and Sn‐3d_3/2_ energy states, respectively. This is affirming the presence of Sn^2+^ state of 1L‐SnS. The corresponding spin‐orbital splitting in Sn‐3d states of ≈8.4 eV is in agreement with that reported in the literature.^[^ ^32^ ^]^ In addition to Sn‐3d states, the S2p peak doublet (with the spin‐orbit splitting of 2p_3/2_‐2p_1/2_ is 1.2 eV) is presented in Figure 2f where the S‐2p_3/2_ binding energy appears at 161.5 eV assigned to SnS chemical state.^[^ ^68^ ^]^ The binding energies for Sn and S core levels obtained for the FL‐SnS flakes were found to be similar (Figure S6, Supporting Information), and the Sn over S atomic ratios (Sn:S) are close to 1:1 and presented in Table S3, Supporting Information. Additionally, UV photoelectron spectroscopy (UPS) measurements have been employed to probe the electronic properties of isolated SnS nanosheets (Figure S6, Supporting Information). The corresponding work function, obtained by linear extrapolation of the lower kinetic energy leading edge of the UPS spectrum (Figure S6, Supporting Information), was measured to be 3.9 and 4.1 eV for 1L‐SnS and FL‐SnS, respectively (Table S3, Supporting Information).

The optical bandgap (*E*_g_) of isolated SnS nanosheets was determined from UV–vis extinction spectra. As shown in Figure S7a, Supporting Information, the extinction spectra of the isolated SnS nanosheets exhibit a broad absorption feature in the UV–vis‐NIR spectral range. It is also observed that the absorption edge is slightly shifted towards lower energies for 1L‐SnS compared to FL‐SnS nanosheets. This observation indicates the variation of *E*_g_ upon decreasing the number of layers. Indeed, as determined from the respective Tauc plots, the estimated *E*_g_ of SnS varies from 1.91 to 1.99 eV for FL‐SnS and 1L‐SnS, respectively. The *E*_g_ value of 1L‐SnS (Figure 2g) is close to that determined via theoretical calculations.^[^ ^11^, ^32^ ^]^ It has been well known that the *E*_g_ of bulk SnS is 1.28 eV and shows a sudden increase when the layer number is reduced, which can be attributed to the quantum confinement effect (QCE); this effect becomes more pronounced when the crystal size approaches the excitonic Bohr radius. Considering the effective mass approximation, the excitonic Bohr radius, *R*, of SnS can be calculated from the following equations^[^ ^69^ ^]^

(2) $$R=\varepsilon a_{0}\left(\frac{m_{0}}{\mu }\right)$$

(3) $$E=E_{g}+\frac{h^{2}}{8\mu r^{2}}-\frac{1.8e^{2}}{4\pi \varepsilon_{0}\varepsilon r}$$

(4) $$\mu =\frac{m_{e}m_{h}}{m_{e}+m_{h}}$$

where *E*_g_ is the band gap of bulk material, *h* is the Plank's constant, *µ* is the reduced mass of the exciton, *r* is the radius of the material, *e* is the electronic charge, and *ε* is the dielectric constant. For SnS, *E*_g_ is 1.28 eV and *µ* for electron and hole is 0.20 and 0.16 *m*_0_, respectively^[^ ^70^ ^]^ where *m*_0_ is the free‐electron mass. Considering *a*_0_= 0.053 nm and *ε*= 13 for SnS,^[^ ^70^ ^]^ the excitonic Bohr radius of SnS obtained from Equation (2) is ≈4.3 nm. As the thickness of the 1L‐SnS of 0.904 nm is much smaller than this value, the QCE is expected to be stronger in 1L‐SnS, giving rise to a remarkable blue shift in the optical bandgap. In order to validate this optical band transition in 1L‐SnS, micro‐photoluminescence spectra were additionally recorded, showing a single broad emission peak centered at ≈1.95 eV, that is, close to the *E*_g_ value obtained from the Tauc plot.

**Figure 3:**
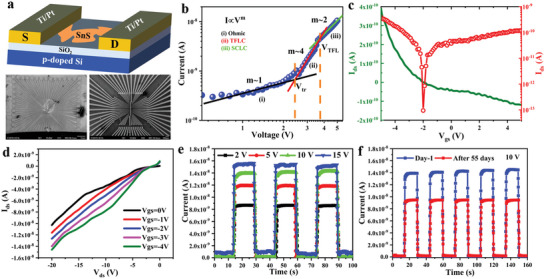
Device structure and electrical characteristics: a) Schematic and SEM images of the device; b) *I–V* characteristics and conduction mechanisms in Au/1L‐SnS/Au device. Both axes are in log scale. Solid symbols are the experimental data, whereas the solid lines correspond to the *I* ∝ *V* ^m^ fittings; c) The transfer characteristic (I_ds_–*V*_gs_) curve of an SnS FET device comprising a Cr/Au contact (right axis in log scale); d) Output characteristic curve (*V*_ds_‐*I*_ds_) of the SnS device; e) Voltage‐dependent characteristics of the Pt/1L‐SnS/Pt device photoresponse under illumination. The incident UV light power is 2.055 mW; f) Environmental stability of the SnS device exposed to air for 2 months. The excitation wavelength is 254 nm (4.88 eV).

In order to investigate the electronic properties of an isolated 1L‐SnS, field effect transistor (FET) devices have been fabricated. The metal gold (Au) electrodes are used as a source and drain. The SnS layer was delaminated onto the pre‐patterned SiO_2_ substrate as described in the Experimental Section. **Figure** **3**a represents a 3D schematic of the devices and the scanning electron microscope (SEM) images of the pre‐patterned electrodes. The AFM and SEM images of the fabricated devices are presented in Figure S8, Supporting Information. Both microscopic images indicate that the SnS sheets are well connected with the metal electrodes.

The current‐voltage (*I–V*) characteristics of the best‐performed and reproducible 1L‐SnS devices are represented in Figure S9a, Supporting Information. The *I–V* characteristic of a two‐terminal device is exhibited as symmetric in nature. The obtained symmetric nature of *I‐V* characteristic infers the absence of the Schottky barrier (SB) and the formation of ohmic contact between the metal electrodes and 1L‐SnS. The polarization property was investigated with cyclic *I–V* measurements. The cyclic *I–V* characteristic exhibits a nonlinear characteristic with a significant hysteresis (see Figure S9b, Supporting Information). The relatively large current at zero voltage ensures a typical resistive switching effect existing in the device.^[^ ^71^ ^]^ On the contrary, the audited phenomenon of such big hysteresis is probably due to the ferroelectric nature in ultrathin layered SnS.^[^ ^20^, ^24^ ^]^ Nonetheless, the presence of traps in the SnS crystal and the trap density near the metal/semiconductor junction cannot be excluded.^[^ ^21^, ^72^ ^]^

In order to explore the underlying charge carrier transport mechanism in 1L‐SnS, the logI‐logV characteristics of the device are plotted (Figure 3b). The logI–logV plot exhibits three distinct regions noted as i, ii, and iii. All regions exhibit different slopes, and the slope change points are marked by the dashed lines in Figure 3b. The obtained transition points are crossover log(voltages) are 2.4 and 3.8 V corresponding to regions i ii and ii iii, respectively. The former crossover voltage (region i ii) defines the transition voltage (*V*_tr_) and the latter (region ii iii) the trap‐filled‐limited voltage (*V*_TFL_). Namely, the *V*_tr_ is the transition cross‐over voltage from thermionic emission (TE) to Fowler–Nordheim tunneling (FNT), while *V*_TFL_ is a transition from FNT to space charge limited conduction (SCLC). It can be assumed that at applied voltage *V* < *V*_tr_, the electrons are lightly injected from the metal electrode (Au) to SnS and the conduction is governed by the thermionic emission over the Schottky barrier. Practically, at low voltage region (region i, *V* < *V*_tr_), the *I–V* characteristic follows the Ohmic conduction mechanism and Ohm's law (see Figure S10, Supporting Information). More specifically, in the Ohmic region, the charge carrier transit time (*τ*_r_) is larger than the dielectric relaxation time (*τ*_d_), and as a result, the density of lightly injected charge carriers (at low bias voltage) in SnS by TE is smaller than the thermally generated carriers.^[^ ^73^, ^74^ ^]^ Moreover, to maintain charge carrier neutrality, the low‐density charge carriers are distributed over the high‐density of thermally generated charge carriers. The injected low‐density charge carriers cannot transport in this conduction regime. At this low applied bias voltage or region i, the logI–logV characteristic follows the Ohmic conduction, which is governed by the relation^[^ ^74^, ^75^ ^]^

(5) $$I=\frac{qn\mu V}{d}$$

where, *I* is the current, *q* is the electronic charge, *n* is the charge density, *µ* is the mobility of the charge carrier, *V* is the voltage, and *d* is the sample thickness.

However, with increasing the voltage of the device, a strong charge carrier injection by FNT appears at *V* > *V*_tr_. In this region, the transit time of injected charge carrier is too short to be relaxed by the thermally generated charge carrier, and as a result, a space charge (SC) field is generated (see Figure S10, Supporting Information). Then the current is controlled by SC and called SCLC. When the trap states are absent or do not affect the injected charge carrier, the current density follows the Mott–Gurney's or Child–Langmuir, *J*∞V^2^, law

(6) $$J=\frac{9}{8}\varepsilon_{0}\varepsilon_{r}\mu \frac{V^{2}}{d^{3}}$$

where, *ε*_0_ and *ε*_*r*_ are the free space and relative permittivity, respectively. However, in the presence of trap states, some of the injected charge carriers are trapped, which limits the charge carrier transport. When this trap is exponentially distributed in energy (see Figure S10, Supporting Information), the current density is given by Mark and Helfrich equation^[^ ^75^, ^76^ ^]^

(7) $$J=\left(\mu \frac{N_{v}}{q^{l-1}}\right){\left(\frac{2l+1}{l+1}\right)}^{\frac{1}{l}}{\left(\frac{\varepsilon_{0}\varepsilon_{r}}{N_{t}}\cdot \frac{l}{l+1}\right)}^{l}.\frac{V^{l+1}}{d^{2l+1}}$$

where, *µ* is the electronic mobility, *N*_v_ is the effective density of states, *q* is the electronic charge, and *N*_t_ is the trap density.

In particular, the *I–V* characteristic follows the universal power law of form *I* ∝ *V* ^m^ with exponent *m*. Where *m* can be determined from the slope of the logarithmic plot (Figure 3b). It is observed that the slope of the *I–V* characteristic curve is varying with applied voltage. The finite slope at region i is close to 1 and follows the Ohmic conduction, while the slopes are increasing with applied voltage. By increasing the applied voltage *V* > *V*_tr_ , the slopes appeared to be > 2. In this region, the injected electrons are increasing, which moves the Fermi level of SnS above the trap level, and as a result the trap states gradually start to fill. This process continues until the trap states are totally occupied by the electrons. The voltage at which all the traps are filled is known as *V*_TFL_. At this voltage, the transition of trapped to trap‐free characteristic appears. With *V* > *V*_TFL_ all the traps are filled with the highly injected electrons and then the subsequent charge carriers are free from traps to move in SnS. The current conduction in this regime is governed by the SCLC, which follows the Mott–Gurney's law, *J* ∞V^2^ (Equation (6)).

Figure 3c represents the transfer characteristic of the 1L‐SnS FET device. Notably, the transfer characteristic of the device infers the preferential *p*‐type carrier transport, since an offset *I*_ds_ current is obtained at *V*_gs_ equal to zero. Moreover, the corresponding output characteristics (*V*_ds_–*I*_ds_) of the device are shown in Figure 3d. The *V*_ds_‐*I*_ds_ characteristic demonstrates the ability to modulate the 1L‐SnS channel resistance for negative gate bias voltage. In particular, the channel resistance of the device is decreasing with increasing the negative gate bias voltage, while consequently the *I*_ds_ of the device is increasing. A current on‐off ratio exceeding ≈10^4^ order for the hole is recorded. Furthermore, the field effect mobility is extracted by using the following equation^[^ ^77^ ^]^

(8) $$\mu =\left(\frac{L}{W\times C\times V_{\mathrm{ds}}}\right)\frac{dI_{\mathrm{ds}}}{dV_{\mathrm{gs}}}$$

where, *L* is the channel length, *W* is the width of the channel, *C* is the specific capacitance of the gate dielectric, and $\frac{dI_{\mathrm{ds}}}{dV_{\mathrm{gs}}}$ is the slope of the *I*_ds_–*V*_gs_ curve. The hole mobility at room temperature is obtained to be ≈0.886 cm^2^ V^−1^ s^−1^ from the linear region of the *I*_ds_/*V*_gs_ plot (Figure 3c). It is noted that the relatively low mobility of the SnS sheets observed in FET devices could be due to the adsorption of the solvent molecules, which stuck to and beneath the sheet, interrupting the ideal interface for transport. Moreover, SnS is prone to surface oxidation, which is detrimental to electronic properties.^[^ ^36^ ^]^ Therefore, the negative role of surface oxidation in the low mobility measured cannot be ruled out.

The photoresponse of the isolated monolayer SnS was systematically explored to further unleash the impressive optoelectronic application. The details of the device fabrication methods are described in Note S1, Supporting Information. Figure S11a, Supporting Information presents a schematic of the optoelectronic measurement setup under the illumination of UV light sources. Figure S11b, Supporting Information illustrates the *I–V* characteristics of the SnS device under illumination. In contrast to the dark condition, the photocurrent of the device increases significantly under illumination with both 254 nm (4.88 eV) and 405 nm (3.06 eV) light sources. Irradiation with the latter source has a more profound effect on the obtained *I–V* features (Figure S11b, Supporting Information). The observed photocurrent increment signifies the contribution of photogenerated charge carriers in the irradiated SnS sheets. The photocurrent is calculated by the equation *I*_photocurrent_ = *I*_illumination_ − *I*_dark_. In particular, the light on/off ratio is defined as the ratio of the *I*_photocurrent_ and *I*_dark_. In order to study the stability and response time, a time‐dependent photoresponse of the devices was measured. Figure S11c, Supporting Information represents the time‐dependent photoresponse of the SnS device with 254 nm illumination at zero bias. The current of the device switches rapidly upon placing on/off the light source. The current in the device is switched from 10^−14^ A (dark) to 10^−10^ A (illumination). A significantly high on/off ratio of over 10^4^ can be achieved, indicating the excellent and ultrahigh sensitivity of the SnS sheets. Remarkably, the obtained value is significantly higher than in other 2D materials with no applied bias voltage. In addition, the photoresponse time constant is defined by fitting with *y* = *Aexp*( ± *x*/*τ*) + *γ*_0_ the different time‐dependent photoresponse characteristics. Namely, it exhibits a rapid photocurrent generation and quenching speed with a time scale of a few tens to hundreds of microseconds. Also, it is found that the growth time is 77 µs, while the decay time is 633 µs with 254 nm illumination at 0 V applied bias.

Moreover, the bias voltage‐dependent switching characteristics of the devices are acquired by changing the bias voltages from 2 to 15 V (Figure 3e and Figure S11d, Supporting Information). The photocurrent rises and decays smoothly upon the on‐and‐off condition of the light illumination varying with applied bias voltages. This observation demonstrates that the fabricated SnS‐based device has good stability and repeatability as a photodetector. Also, a linear increase in the photocurrent was recorded. The recorded current is 8.5 × 10^−9^, 1.19 × 10^−8^, 1.39 × 10^−8^, and 1.55 × 10^−8^ A (254 nm illumination) at 2, 5, 10, and 15 V, respectively. While the recorded current is 1.08 × 10^−11^ A (2 V), 1.26 × 10^−11^ A (5 V), 1.42 × 10^−11^ A (10 V), and 1.64 × 10^−11^ A (15 V), under 405 nm illumination. In both cases, the photocurrents increase linearly with applied bias voltage. This observation confirms that the photoresponse of the device can also be tuned in accordance with the applied bias voltage. Notably, in the monolayer devices, the photoresponse reveals a rapid photocurrent generation and quenching speed with a time scale of a few hundred microseconds. It is acquired that the growth time is 401 µs, while the decay time is 390 µs with 254 nm illumination (**Table** **1**). On the contrary, the growth and decay time is recorded to be 387 µs and 494 µs with 405 nm illumination.

**Table 1:** Comparison of key parameters of our work based on percolated monolayer SnS film with previously reported other anisotropic 2D materials‐based photodetectors (single flake devices)

Materials	Fabrication method	Flake thickness [nm]	Wavelength [nm]	Response time	Responsivity [R = I_p_/PS]	Ref.
BP	LPE	4‐8	360	0.5 s	1.9‐2.2 µA W^−1^	[78]
GeS	CVD	20‐50	530	0.85 s	139 A W^−1^	[79]
GeS	Solution based	160	350	0.11 s	173A W^−1^	[80]
GeSe	Solution based	250	405	0.2 s	43.6–76.3 µA W^−1^	[80]
GeSe	Solvent or mechanical cleavage method	4.3	350	0.2 s	43.6–76.3 µA W^−1^	[81]
GaS	Mechanical cleavage	‐	254	< 30 ms	19.2 A W^−1^	[82]
GaSe	PVD	Few layer	254	20 ms	2.8 A W^−1^	[83]
SnS	Liquid metal	monolayer	650	0.12	920 A W^−1^	[32]
MoO3‐x	PVD	8 nm	365	0.2 ms	54.4 mW A^−1^	[57]
PdSe_2_	Self‐flux and subsequent mechanical exfoliation	Few layer	369	3.2 s	14.5 A W^−1^	[84]
NiPS_3_	CVD	4.7 nm	254	20.7 ms	120 µA W^−1^	[85]
SiAs	Solid‐state reaction and subsequent mechanical exfoliation	21 nm	325	3 s 1.5 s	16 mA W^−1^	[86]
1L‐SnS	T‐LPE	0.90	254	0.40 s	6.7 µA W^−1^	Present work
1L‐SnS			405	0.38 s		

In order to evaluate the performance of the photodetector, the photoresponsivity (PR) of the device was calculated. The PR was obtained by *R* = *I_ph_*/*P_in_S*, where *I_P_*_h_ is the photocurrent, *S* is the active area, and *P*_in_ is the incident power. The responsivity was calculated to be 6.7 µA W^−1^ under a light intensity of 4 mW cm^−2^. It is worth mentioning that the responsivity of the device is high upon UV illumination and comparable with those of the reported anisotropic 2D materials‐based photodetectors (Table 1). In particular, the PR of our device is higher than in the isostructural and isoelectronic BP. We believe that, following their deposition from the solution, the self‐arrangement of SnS monolayers forms a percolation path for the photogenerated charge transport. This percolation can favor the hopping charge carrier transport mechanism, from one layer to the next, rather than the band‐to‐band transport one, thus limiting the photoresponsivity. Therefore, due to its percolated nature, the PR of the SnS film of this study should be lower than that of the best reported (at 650 nm) to date for an SnS monolayer ^[^ ^32^ ^]^. Aiming to shed more light on the origin of the photoresponse behavior of the fabricated SnS devices, we considered and proposed a feasible photoresponse mechanism that relies on charge transfer in SnS and Pt electrodes (shown in Note S3b and Figure S12, Supporting Information).

The photoresponse characteristics of the device to light with different wavelengths were also detected. Figure S11e, Supporting Information represents the photoresponse characteristic of the device in the presence of white light illumination. Most interestingly, the device is only sensitive to deep UV light (254 nm) but fully blind to white light illumination. Remarkably, at the same time, the dark current is invariant to illumination of white light. Such findings unambiguously demonstrate the promising potential of 1L‐SnS as a large‐scale solar‐blind deep UV photodetection.

Finally, the stability of the device was tested and verified by measuring the photoresponse after a couple of months. Figure 3f presents the photoresponse profiles of the SnS‐based device after storing at lab‐ambient atmosphere. Apparently, the photoresponse behavior of the device under a light on and off condition remains similar. However, a slight drop in the device photocurrent was recorded. In particular, the obtained photocurrent is recorded to be 1.4 × 10^−8^ A (at 10 V) on the first day, while dropping to 1.0 × 10^−8^ A after 2 months. Such a drop in the photocurrent may be considered negligible in terms of the photoresponse operation. Most importantly, the response and recovery time of the device remain the same. These experimental results immensely suggest that the device is quite environmentally stable for long‐term applications.

In summary, we have demonstrated a new LPE approach for the efficient isolation of high‐volume and high‐crystallinity monolayers of SnS. This is based on the synergy of external driving energies due to ultrasonic hydrodynamic cavitation and heating. It is shown that such synergy significantly impacts the isolation of 1L‐SnS and is capable to overcome the strong interlayer binding energy among the SnS crystal lattice planes. Fine analysis, based on non‐linear optical microscopy, revealed the in‐plane structural anisotropy of the exfoliated crystals, along armchair and zig‐zag directions. Microelectronic devices fabricated from solution showed that SnS single‐layer crystals exhibit appreciable carrier mobility and deep‐UV spectral photodetection, with high response time and photoresponsivity. At the same time, monolayer‐based SnS transistor devices presented a high on/off ratio and remarkable stability upon prolonged operation. This study opens a new avenue for large‐scale isolation of highly crystalline SnS and other MMC nanolayers for a wide range of applications, including extended area nanoelectronic devices, or wearable emerging electronics, photonics, and optoelectronics, printed from solution via industrially compatible processes including spin and spray coating ones. At the same time, the intriguing intrinsic optoelectronic properties of MMC monolayer crystals at their quantum limits could open up an opportunity for fundamental science in future quantum electronics applications.

## 3 Experimental Section

### Synthesis of SnS Monolayer

The liquid phase exfoliation method was employed for the isolation of a monolayer SnS. In the LPE method,^[^ ^39^, ^43^ ^]^ tin (II) sulfide granular (> 99.99%, Lot no‐40105, Sigma Aldrich, USA) trace metals basis (3 mg ml^−1^) was dissolved in acetone (≥ 99.5%, Honeywell, Germany) in nitrogen (N_2_) flushed glass vials and sealed with Teflon‐tape with nitrogen. The cavitation in solution was created with a bath ultrasonication (Elma S 30 H, Elma Schmidbauer GmbH, Germany), using a power of 80 W and a frequency of 37 kHz for 10 and 20 h. During sonication, the bath temperature was raised up to 50 °C, that is, below the boiling point of acetone (at 56 °C). Following the sonication process, a dark brown solution was obtained, which was centrifuged at 5000, 8000, and 12 000 rpm for 15 min. The optimum centrifugation speed for the 20 hcavitated sample was found to be 8000 rpm for 15 min. In order to investigate the temperature‐dependent LPE, the results were compared with dispersions obtained upon using a bath temperature of < 25 °C, during the ultrasonication process.

### Characterization of SnS Monolayers: AFM

The surface topography of isolated SnS was analyzed by AFM (Digital Instruments, USA) in tapping mode with controller Nanoscope IIIa. The isolated dispersion of SnS was drop‐casted on a pre‐cleaned Si substrate and vacuum dried before measurement. The vacuum‐dried sample was placed on the AFM stage to scan. The AFM imaging was performed at ambient conditions, that is, a temperature of 25 ± 1 °C, and relative humidity of 50–60%. Pertinent scanning parameters were as follows: scan rate for all measurements – (1.08 Hz for Figure 1b), (1.70 Hz for Figure 1c); aspect ratio: 1:1; resolution: 512 samples/line, 512 lines. The FFT images are analyzed in NanoScope Analyses 1.5 software, Bruker, USA.

### Characterization of SnS Monolayers: HRTEM

Atomic‐scale structural analysis of the nanoflakes was performed using a high‐resolution TEM/STEM Cold Field Emission Gun (CFEG) JEOL JEM F200 (https://www.jeol.co.jp/en/products/detail/JEM‐F200.html) electron microscope operated at 200 kV, with HRTEM point‐to‐point resolution of 0.19 nm and a spherical aberration coefficient *C*s = 0.5 mm. HRTEM images were acquired by a bottom‐mounted GATAN RIO (https://www.gatan.com/products/tem‐imaging‐spectroscopy/rio‐camera) 9 Mps CMOS camera, using the GATAN Digital Micrograph Suite (https://www.gatan.com/products/tem‐analysis/gatan‐microscopy‐suite‐software). Sample preparation included a carbon‐coated copper grid, pinched by a tweezer and dropped in liquid phase SnS solution. Copper grids were left to dry without any surface contact for 8 min before being left for an additional hour under a visible light lamp to completely dry. Image Processing and Analysis were performed using the GATAN Microscope Suite 3. The balls and sticks model was built whereas the simulated SAED was constructed using the CrystalMaker (http://crystalmaker.com/about/index.html) and the SingleCrystal (http://crystalmaker.com/about/index.html) software packages respectively. Through thickness‐defocus HRTEM image simulation maps were performed using the JEMS software package (https://www.epfl.ch/research/facilities/cime/research/research‐jems/).

### X‐Ray and UV Photoelectron Spectroscopies

The X‐Ray and UV photoelectron spectroscopy measurements (XPS/UPS) were performed in a UHV chamber (P ≈5 × 10^−10^ mbar) equipped with a SPECS EA10 hemispherical electron analyzer, a non‐monochromatized dual‐anode Mg/Al X‐ray gun for XPS and a UV source (model UVS 10/35) produced HeI irradiation with *hν* = 21.22 eV. The XP Spectra were recorded with MgKa at 1253.6 eV photon energy and an analyzer pass energy of 36 eV giving a full width at half maximum (FWHM) of 1.7 eV for Ag3d_5/2_ line. The analyzed area was a spot of 3 mm in diameter. The XPS core level spectra were analyzed using a fitting routine, which can decompose each spectrum into individual mixed Gaussian‐Lorentzian peaks after a Shirley background subtraction. The atomic ratios were calculated from the intensity (peak area) of the XPS peaks weighted with the corresponding relative sensitivity factors (RSF). During the UPS measurements, the samples were biased at −12.3 V to avoid interference with the spectrometer threshold in the UPS spectra and observe the secondary electron edge where the work function of the surface can be determined. The work function can be estimated by subtracting the secondary electron cutoff from the He excitation source of 21.22 eV (Φ = 21.22 eV − E_SEC_).

### Raman Spectroscopy

Raman spectroscopy measurements were performed at room temperature using a Nicolet Almega XR µ‐Raman analysis system (Thermo Scientific Instruments, Waltham MA USA), in backscattering geometry, equipped with an air‐cooled solid‐state laser operating at 473 nm. The laser beam was focused using a 50X long‐working distance objective with  1800 g mm^−1^ grating on the sample. The samples were prepared by drop casting dispersion of SnS sheets on cleaned Si substrates. The spectra were collected after calibrating the spectrograph with Si substrate (peak located at 520 cm^−1^ was taken as an internal reference).

### Optical Spectroscopy

The optical UV–vis extinction spectra of the dispersions were carried out with a PerkinElmer, Lamda 950 UV/VIS/NIR spectrometer, USA. All measurements were carried out with all dispersion of isolated SnS in acetone using a quartz cuvette (path length of 10 mm).

### Steady‐State µ‐Photoluminescence Spectroscopy

For optical spectroscopy measurements at 300 K, a micro‐photoluminescence (µ‐PL) setup was used and the spectra were collected in backscattering geometry. The excitation source used is a 543 nm (2.28 eV) CW laser. A Mitutoyo 50x focuses down to ≈1 µm the spot size for the sample excitation. An iHR‐320 spectrometer (Horiba Scientific/Jobin Yvon Technology) was used to collect the spectra. The sample was produced by drop casting on a pre‐cleaned SiO_2_ substrate and dried under vacuum. The PL measurements are performed under an ambient atmosphere.

### FET Device Fabrication and Characterizations

The exfoliated 1L‐SnS flakes are delaminated onto a 290 nm thick SiO_2_ on Si substrate that was pre‐patterned with a multi‐electrode array. The multi‐electrode array was fabricated via electron beam lithography. In particular, a set of 56 optically defined pads (AZ2020, MA6 contact lithography, Cr 2 nm / Au 100 nm) was deposited using lift‐off. The inner side of the conductive lines forms a rectangular area, 100 × 100 µm wide, where electron beam lithography was used to define nanoelectrodes (A4 PMMA, JEOL 7000F with RAITH Quantum e‐beam writer at 30 KV, 2 nm Cr / 20 nm Au) of varying gap from 10 nm to 5 µm. Prior to the use of the exfoliated SnS nanosheets, the isolated dispersion was diluted. A tiny amount (2 uL) of SnS dispersion was cast on the above‐mentioned substrate with the help of a fine pipette. The casted sample was left for natural drying and after several hours the sample was dried under vacuum overnight. The sample was annealed at 60 °C for 15 min to improve the electrical contact and remove the chemical residue. Finally, the device was used for electrical measurements. The electrical measurements of FET devices were carried out using the Keithley 4200 semiconductor parameter analyzer (Keysight) in a probe station. All measurements were performed under an ambient atmosphere.

## Conflict of Interest

The authors declare no conflict of interest.

## Supporting information

Supporting Information

## Data Availability

The data that support the findings of this study are available from the corresponding author upon reasonable request.
