# Supplementary material for: Liquid Phase Isolation of SnS Monolayers with Enhanced Optoelectronic Properties
Source: Adv Sci (Weinh). 2022 Dec 27;10(6):2201842. doi: 10.1002/advs.202201842 (PMC9951343; doi:10.1002/advs.202201842)
Supplement: Supplementary file 1 — Supporting Information [file ADVS-10-2201842-s001.pdf]

Supporting Information

Liquid phase isolation of SnS monolayers with enhanced optoelectronic properties

A. S. Sarkar*[1], I. Konidakis[1], E. Gagaoudakis[1], G. Maragkakis[1,2], S. Psilodimitrakopoulos[1], D. Katerinopoulou[1,2], L. Sygellou[3], G. Deligeorgis[1], V. Binas[1,2], I. P. Oikonomou[4], Ph. Komninou[4], G. Kiriakidis[1], G. Kioseoglou[1,5], E. Stratakis*[1,2]

[1]Institute of Electronic Structure and Laser, Foundation for Research and Technology-Hellas, Heraklion, 700 13 Crete, Greece.

[2]Physics Department, University of Crete, Heraklion, 710 03 Crete, Greece.

[3]Institute of Chemical Engineering Sciences (ICE-HT), Foundation of Research and Technology, Hellas, P.O. Box 1414, 26504 Rio Patras, Greece

[4] Department of Physics, Aristotle University of Thessaloniki, Thessaloniki, 54124, Greece

[5] Materials Science Department, University of Crete, Heraklion, 710 03 Crete, Greece.

Abbreviations

ME: Mechanical Exfoliation

PVD: Physical Vapor Deposition

MBE: Molecular Beam Epitaxy

ALD: Atomic Layer Deposition

PLD: Pulsed Laser Deposition

LMS: Liquid Metal Synthesis

LPE: Liquid Phase Exfoliation

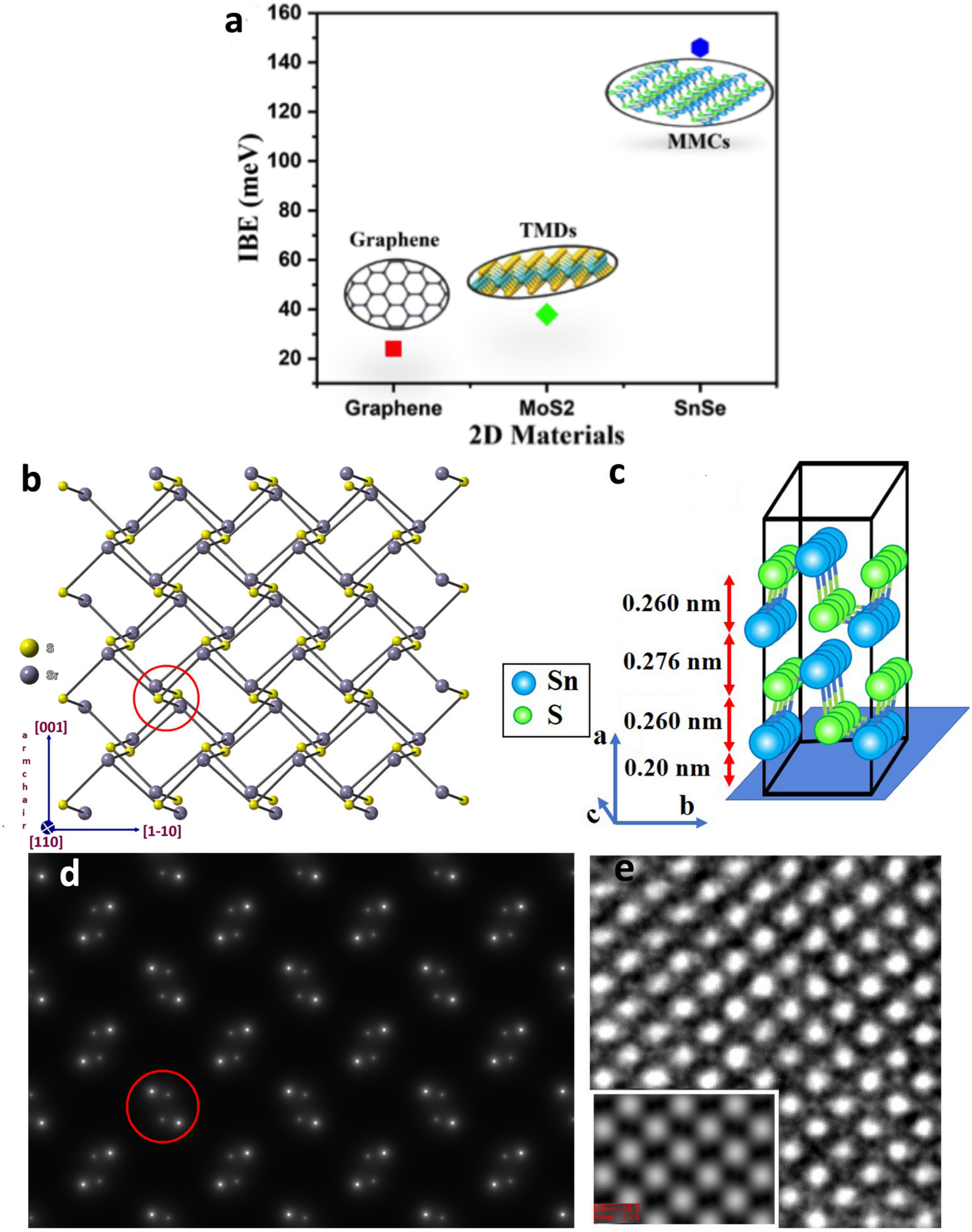

Supplementary Figure S1| Atomic structure of buckled SnS|: a) Comparative plot of interlayer binding energy (IBE) of various 2D materials including graphene, transition metal dichalcogenides (Molybdenum disulfide, MoS_2) and metal monochalcogenide (tin selenide, SnSe)[1]; **b)** Ball and sticks supercell model of the Pnma SnS structure, projected along the

[110] zone axis, used for the simulation of the HRTEM image; **c)** Orthorhombic unit cell of SnS. Two layers of SnS are stacked in a single unit cell; **d)** Simulated image showing the projected potential of the supercell; **e)** Experimental HRTEM image and as inset superimposed the corresponding simulated, for a nanosheet thickness equal to 1.2 nm and a defocus value -60 nm. The group of the four neighboring atomic species (two Sn and two S within the red circle) appear as a bright dot in both the experimental and the simulated images.

Supplementary Table S1. Comparison of synthesis methods adopted for thin layer of SnS

Method	Layer (thickness/number)	Shortcoming /Limitations	Ref.
ME	~10 nm	Dimensional reproducibility<br>Isolation of a single layer<br>Strong interlayer binding energy	[2, 3]
PVD	5.5 nm	High growth temperature at >450°C, substrate suitability	[4]
MBE	Few layers	Ultrahigh vacuum, high growth (450°C) and substrate (200°C) temperature, grown in specific substrates	[5]
ALD	Few layers	Ultrahigh vacuum and high growth temperature (>250°C)	[6]
PLD	Few layers	High substrate temperature, Nonuniform thin films	[7]
Liquid metal	ML	High growth temperature at 350°C, Inert atmosphere, time consuming process, mass production for large scale applications	[8]
Au mediated exfoliations	Multilayer	Formation of oxide layer of SnO_x	[2]

Supplementary Table S2. Summary and comparison of the experimental methods employed to synthesis of 2D SnS.

Synthesis Method	Growth temperature	Average thickness (nm)	Lateral dimension (nm)	Layer number	Ref.
**ME**	-	10 nm	200 nm-1 µm	Multilayers	[2]
**CVD**	Substrate: 420°C	4 nm	Film	multilayer	[9]
**PVD**	Source: 470°C Substrate: 410°C	0.94 nm	1 µm	1L	[10]
**MBE**	Source: 450°C Substrate: 200°C	0.6 nm stacked weeding cake	-	Mono- and few- layers	[5]
**ALD**	240-285°C	-	1 µm	-	[6]
**PLD**	Substrate: 200°C After deposition: 300°C	3.0 nm<br><br>0.7 nm	Uniform Film<br>Nonuniform film	10 L<br><br>2 L	[7]
**LMS**	350°C	0.7 nm	-	1L	[11]
		0.8 nm	-	1L	[8]
**LPE**	Acetone	1.10	170	Bilayer	[12]
**LPE**	NMP	4.2	5-100	6-8 layers	[13]
**LPE**	Acetone	0.90	-	1L	Present work

AFM measurements

The atomic force microscopic measurements were performed at ambient condition. The AFM system was calibrated with a standard sample before the sample scan. Pertinent scanning parameters were as follows: aspect ratio: 1:1; resolution: 512 samples/line, 512 lines. The FFT images are analyzed in NanoScope Analyses 1.5 software, Bruker, USA.

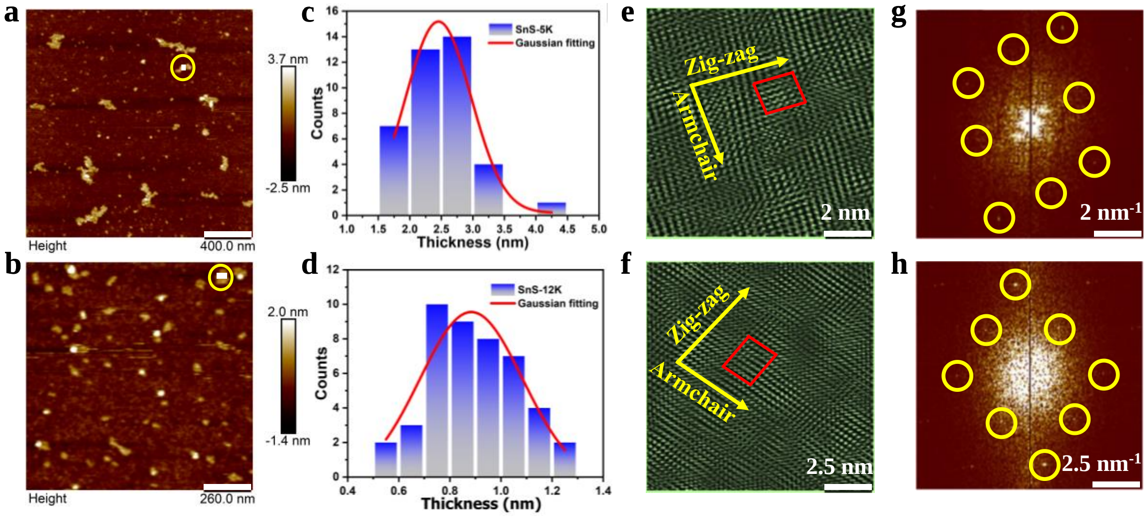

Supplementary Figure S2| Atomic force microscopic characterization of SnS flakes with different centrifugation speed. **a**, **b,** 2D images of SnS with 5K and 12K rpm. **c, d,** Histograms of thickness distribution of SnS with 5k and 12k rpm **e, f,** Fast Fourier transformed filtered atomic resolution of the selected area in **a** and **b**. Scale bar is 2 nm and 2.5 nm in **e** and **f**. **g, h,** Fast Fourier transform of original lattice resolution image of the selected region on SnS flakes. Scale bar is 2 nm^{-1} and 2.5 nm^{-1} in **g** and **h**. The obtained average thicknesses is 2.453±0.057 and 0.883±0.025 for 5k and 12k SnS, respectively.

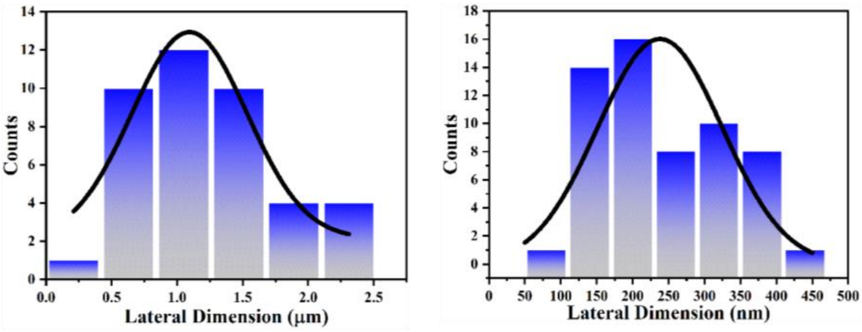

Supplementary Figure S3| Histograms of lateral size distribution for **a** 10h cavitated SnS **b** 20h cavitated SnS. Average lateral dimension is 1.093±0.108 mm and 238±86 nm for 10h and 20h cavitated at 50°C SnS, respectively. **c** schematic of orthorhombic single unit cell of monolayer SnS.

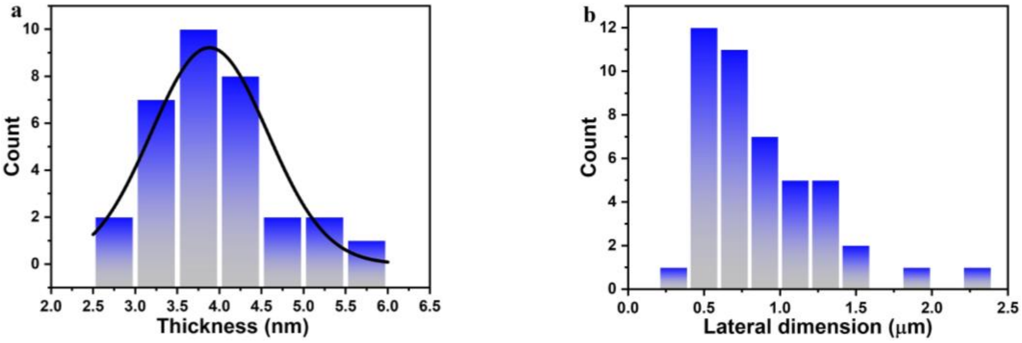

Supplementary Figure S4| Atomic force microscopic characterization of SnS flakes produced at a bath temperature of <25 ^{o}C. Histograms of thickness distribution **a** and lateral dimensions **b**.

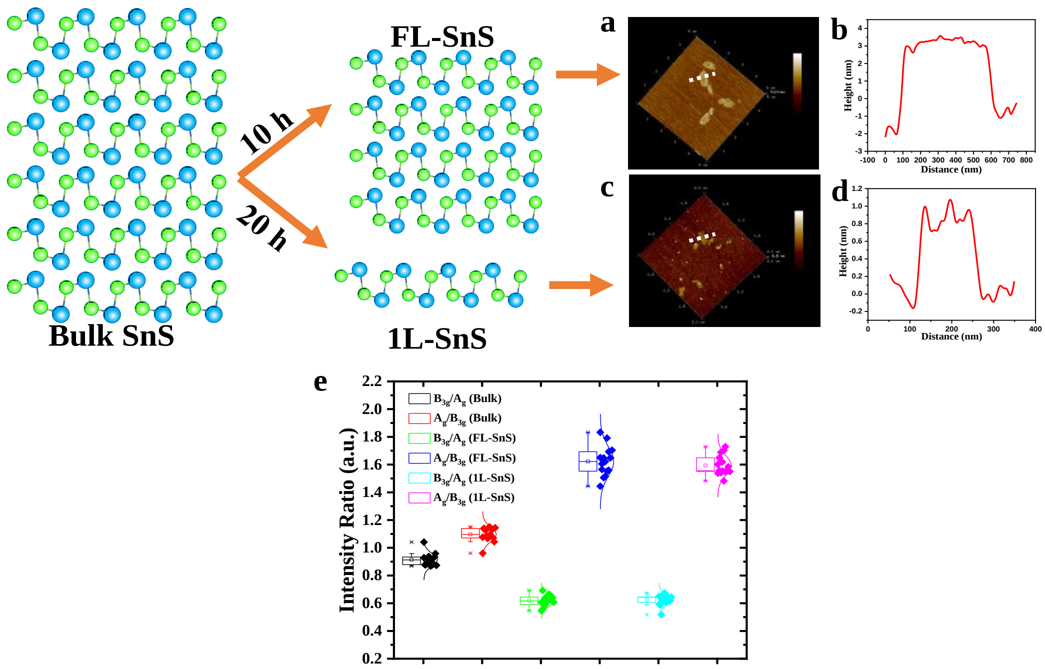

Supplementary Figure S5| Demonstration of layers exfoliation and characterization of SnS. a-b AFM image and height profile of FL-SnS. **c-d** AFM image and height profile of 1L-SnS and **e** Raman spectra of bulk, isolated FL-SnS and 1L-SnS. Four optically active Raman vibrational peaks corresponds to B_{3g}, A_g (1), A_g (2) and A_g (3) modes. **f** Intensity ratio of A_g and B_{3g} Raman modes. A_g/B_{3g} ratio increases with reduction of SnS layer number, while B_{3g}/A_g ratio decreases with reduction of layer number. The Raman spectral mapping were averaged with fifteen measurements in different flakes of SnS.

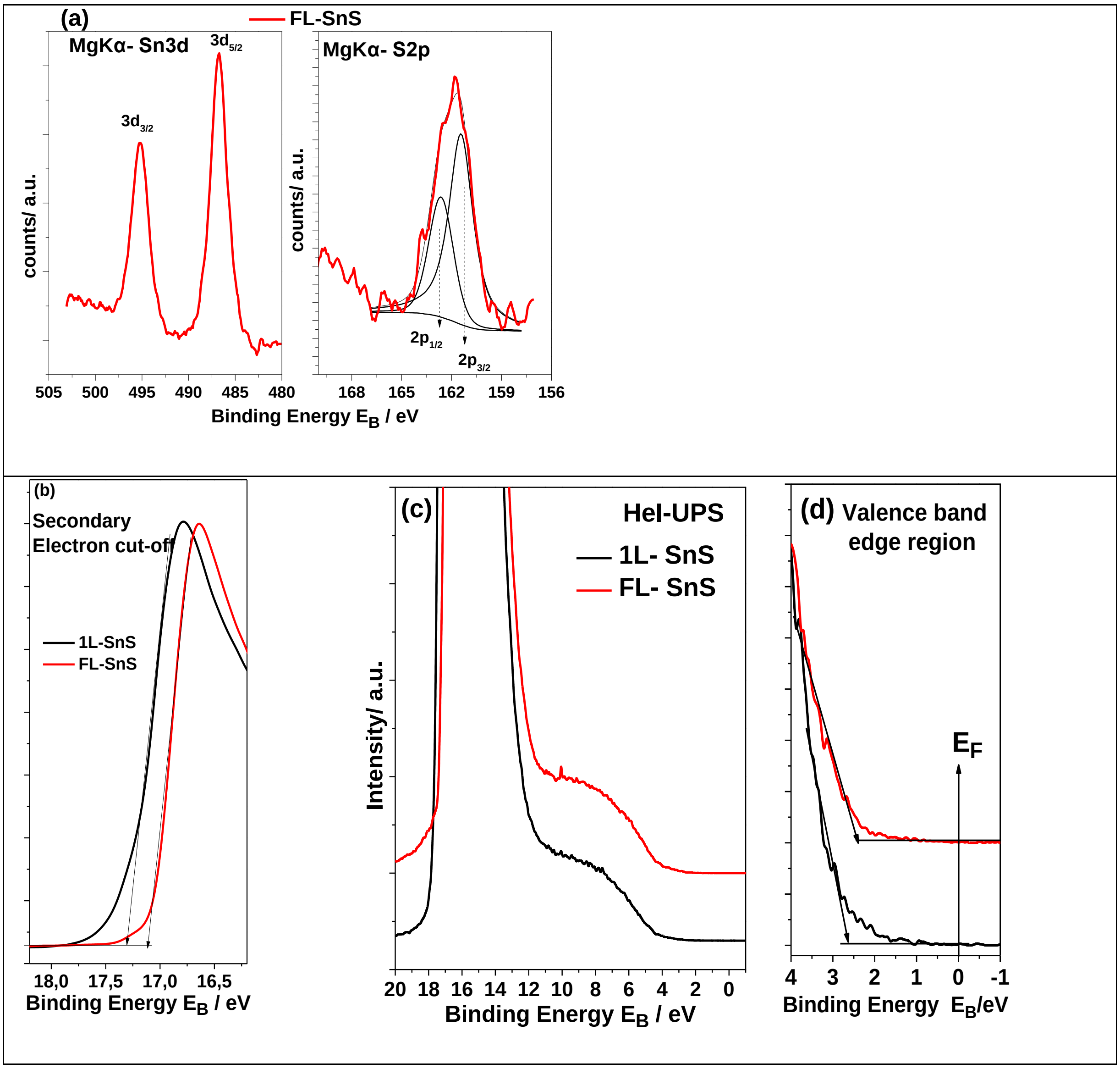

Supplementary Figure S6| X-Ray photoelectron and ultraviolet (UPS) photoelectron spectroscopic characterizations. a S2p and Sn3d detailed spectra of few layer SnS. **b** Secondary Electron cutoff. **c** UPS spectra and **d** close-up of valence band region of monolayer and few SnS layers.

Supplementary Table S3. Sn:S atomic ratio in exfoliated SnS samples

Sample	Sn:S	Work function (eV)	Ionization potential (±0.1) (eV)
1L-SnS	1:0.95	3.9	6.4
FL-SnS	1:1.0	4.1	6.5

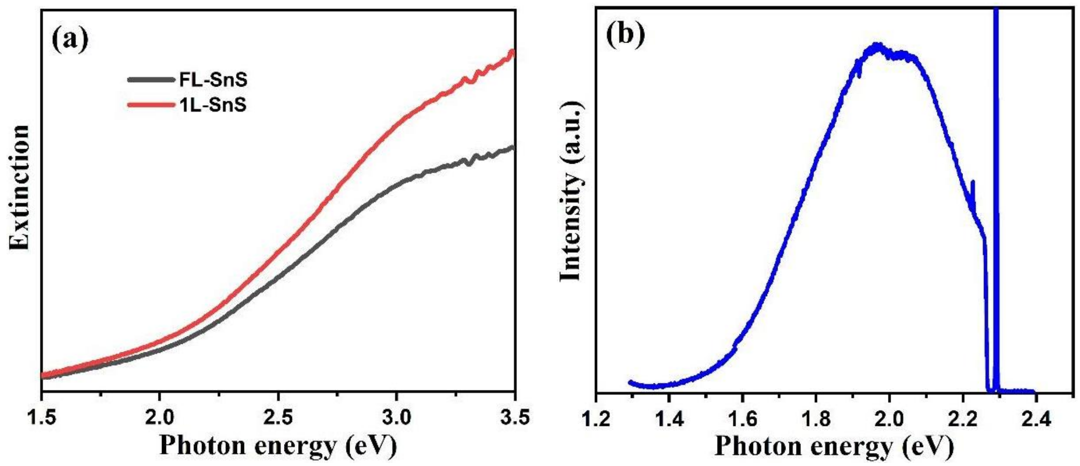

Supplementary Figure S7| Optical properties of isolated SnS. a Extinction spectra of FL-SnS and 1L-SnS. **b** μ-photoluminescence spectra of 1L-SnS. The spectra was collected from the cluster/ensemble of the monolayers SnS. The excitation source was 543 nm (2.28 eV) laser (~1 μm laser spot size) with 640 μW incident power. **c** A representative figure showing the exfoliation of bulk SnS to monolayer SnS. The method displayed the external thermal energy induced liquid phase exfoliation of SnS. Molecular structures are acetone solvent used as an exfoliation medium.

Supplementary Table S4. Summary of current strategies of the experimental LPE methods employed to prepare MXs.

MMCs	Solvent	Sonication length (h)	Average thickness (nm)	Lateral dimension (nm)	Layer number	References
GeS	NMP	1	2.87±0.65	66.91±0.42	6 layers	[14]
GeS	NMP	4	1.3±0.1	-	Tri-layer	[15]
GeSe	Ethanol	56	2	50-200	4 layers	[16]
GeSe	NMP	6	4.3 ± 0.2	-	8-9 layers	[17]
SnS	NMP	24	4.2±0.24	5-100	6-8 layers	[13]
SnS	NMP	56	6	-	Few layers	[18]
SnS	IPA	20	4-8	50	Few layers	[19]
SnS	DMF	1	4.5	400-900	8 layers	[20]
SnS	Acetone	20	1.10	170 (average)	Bilayer	[12]
SnSe	IPA	20	4.3	50-500	7 layers	[21]
SnSe	NMP	2	2.5	50-200	4 layers	[22]

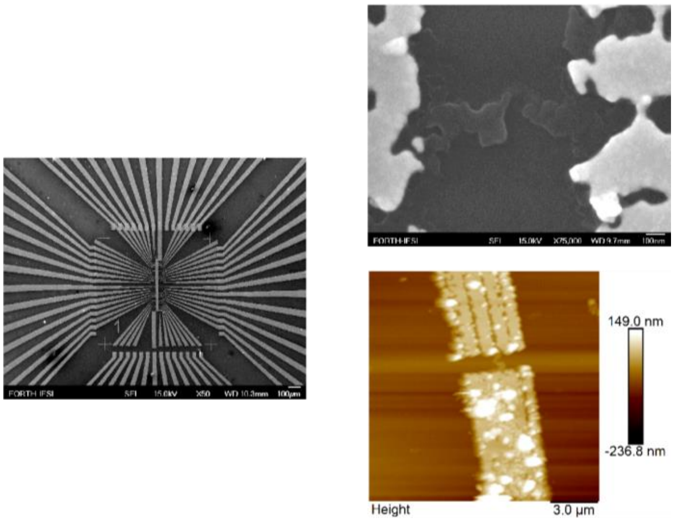

Supplementary Figure S8| Microscopic images of SnS devices. a Scanning electron microscopic image of pattered Au electrodes (variable channel length). **b** Scanning electron microscopic image of Au/SnS/Au device and c Atomic force topography image of Au/SnS/Au device. Height of the Au electrodes are 100 nm. Channel length is ~700 nm.

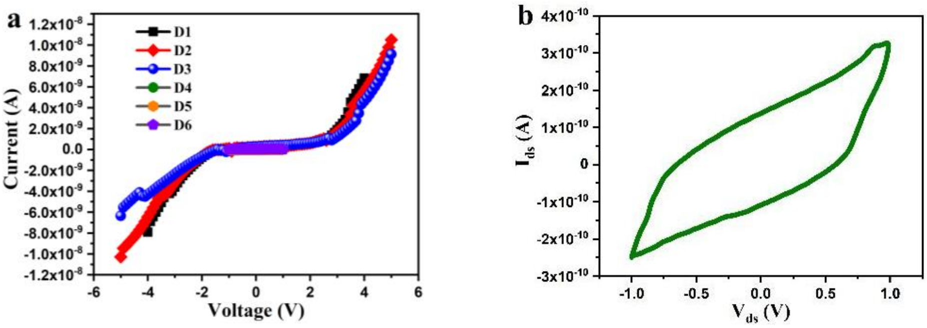

Supplementary Figure S9| Device electrical and microscopic characteristics. a Current-voltage characteristic of monolayer SnS devices **b** Cyclic current-voltage characteristic.

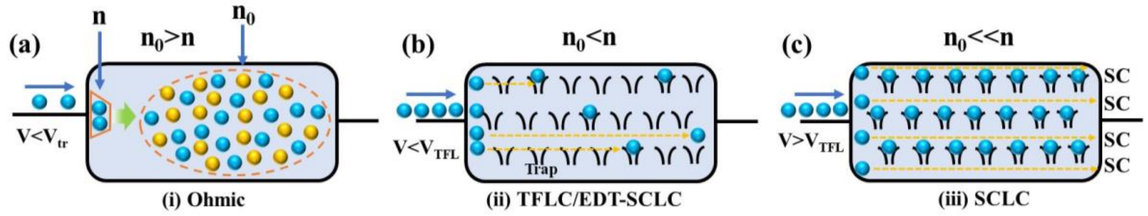

Supplementary Figure S10| Schematic representation of conduction mechanism in SnS| **a** Ohmic transport in Au/SnS/Au at V<V_{tr}, in region i. In this region the carriers are very weekly injected by Thermionic emission. n_0 is thermal carriers and n is injected carriers. **b** trap-filled-limited (TFL)/EDT-space charge limited conduction (EDT-SCLC), in region ii. In this region the carriers are strongly injected Fowler-Norddheim tunneling. The injected carriers is not sufficient to fill all the traps. **c** Space charge limited conduction (SCLC) at V>V_{TFL} in region iii. In this region the the carriers are ultrastrongly injected by Fowler-Norddheim tunneling, as a result all traps are filled by high density of injected charge carriers.

Note 1. Device fabrication

For the photodetector device fabrication pre-patterned interdigitate electrodes (IDE) were used, purchased from ΩMetrohm DropSens, Switzerland (Reference: DRP-G-IDEPT 10). The IDE electrodes are made of platinum contained 2x250 digits with a 5 μm digit gap, while each digit possesses a length of 6760μm and a width of 5μm. Isolated 1L-SnS dispersion solution was used to fabricate the device. The 1L-SnS dispersion was sonicated and deposited on top of the IDEs via drop casting. In particular, a small quantity 50 μL SnS solution was casted with the help of μ-pipette. Following this, the sample was dried for several hours at room temperature, and kept under vacuum overnight. Finally, the device was annealed at 60°C for 15 min to improve the electrical contact and to remove the solvent residues.

Note 2. Device photoresponse measurement and set up

The photoresponse characteristics of the developed devices were measured in a vacuum chamber with a semiconductor parameter analyzer. A UV lamp and a laser diode with wavelength of 254 nm and 405 nm, respectively, were used as illumination light sources. The output laser power of the 405 nm wavelength was electronically controlled. Arduino software application was used to interface and control the laser electrical power and the corresponding laser output. The beam was guided to the sample using focusing lens (10 cm focal length) situated on the top of vacuum chamber (Figure S11). The indoor tube light was used as a white light source. The temporal response of the devices with light illumination was measured with Keithley parameter analyzer. Finally, the effective excitation power on the device which contributes to the photocurrent is obtained as optical power density × illuminated area of the device.

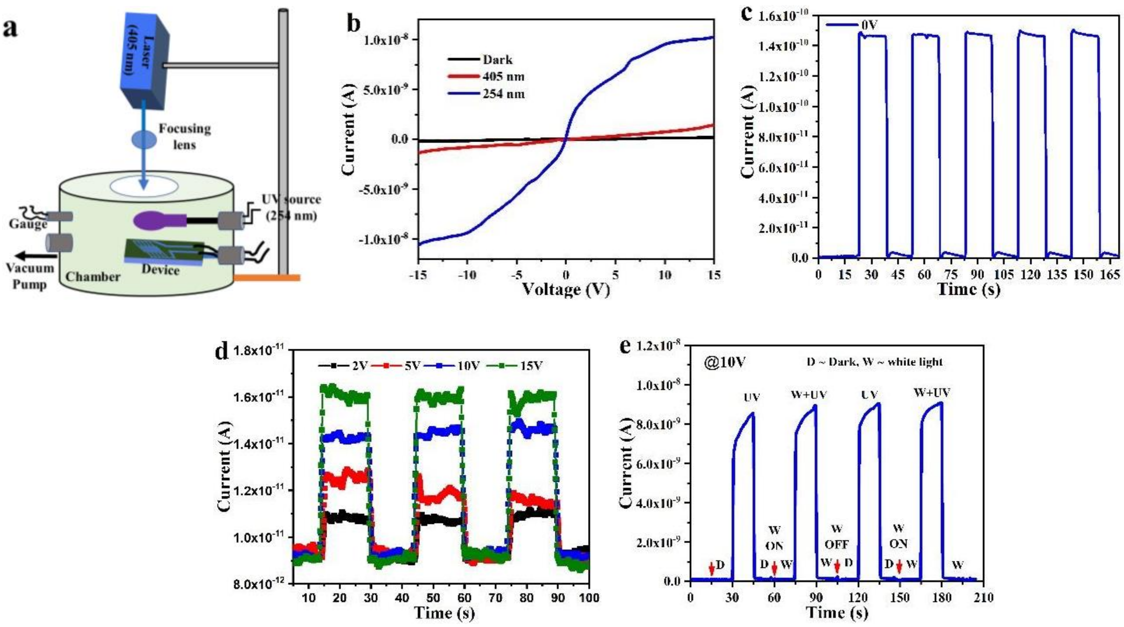

Supplementary Figure S11| a Schematic of the device characterization set up. **b** I-V characteristics of the Pt-1L-SnS-Pt devices under dark and illumination with 254 and 405 nm wavelength. **c** Time-dependent photocurrent response of SnS device under 254 nm light illumination with zero bias voltage. **d** Voltage-dependent photoresponse characteristics of the

Pt/1L-SnS/Pt device under illumination of 405 nm light and **e** Photoresponse I-V characteristics (254 nm illumination) of SnS device in the presence of white light.

Note 3. Photoresponse mechanism

In order to understand the photoresponsive behavior of the developed devices, we consider the energy band diagram of the Pt and SnS (Figure S12). In particular, **Figure S12b** represents the energy band diagram of the Pt-1L-SnS device under dark condition. In this state, when Pt and SnS come into contact a junction is formed as a result of the band bending. In particular, a Schottky-like junction can be formed at Pt-SnS interface due to the difference in work functions of Pt (5.26 eV) and 1L-SnS (4.1 eV). As a result of this, a built in potential would be developed at the interface. This potential leads to the separation of photogenerated charge carrier (either electron or holes) at the interface. When the UV lights falls on the device, the excitons (positive and negative charge bounded by electrostatic force or electron-hole pairs) are generated in SnS layers. This electron-hole pairs are separated at the Pt-SnS interface. This photogenerated charge dissociation/separation induced by the potential results in further band bending at the interface. Consequently, the dissociated charge carriers are transferred towards the Pt electrodes and an increase in the measured photocurrent appears.

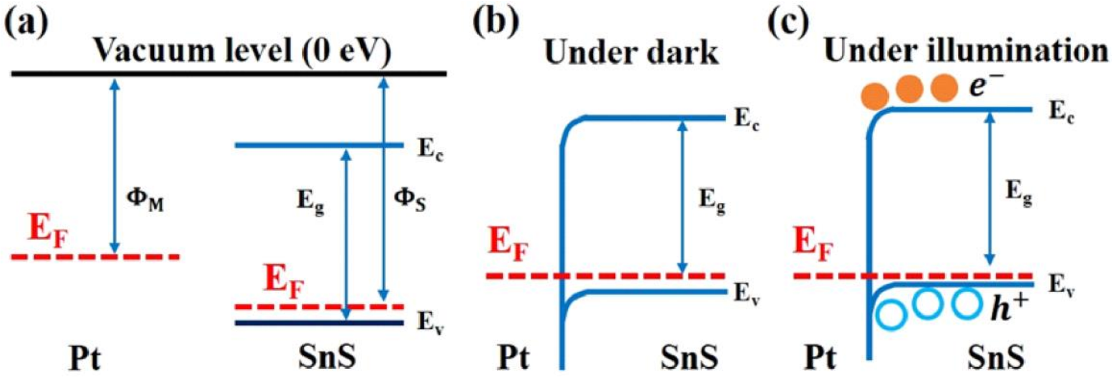

Supplementary Figure S12| Schematic energy band diagram of the SnS photodetector (**a**), before metal-semiconductor junction, **(b)** under dark condition, and **(c)** under illumination.

References

[1] H. Y. Song, J. T. Lu, *Chem. Phys. Lett.* **2018**, *695*, 200.

[2] N. Higashitarumizu, H. Kawamoto, K. Ueno, K. Nagashio, *MRS Adv.* **2018**, *3*, 2809.

[3] N. Higashitarumizu, H. Kawamoto, M. Nakamura, K. Shimamura, N. Ohashi, K. Ueno, K. Nagashio, *Nanoscale* **2018**, *10*, 22474.

[4] J. Xia, X. Z. Li, X. Huang, N. Mao, D. D. Zhu, L. Wang, H. Xu, X. M. Meng, *Nanoscale* **2016**, *8*, 2063.

[5] Y. Bao, P. Song, Y. Liu, Z. Chen, M. Zhu, I. Abdelwahab, J. Su, W. Fu, X. Chi, W. Yu, W. Liu, X. Zhao, Q.-H. Xu, M. Yang, K. P. Loh, *Nano Lett.* **2019**, *19*, 5109.

[6] I. H. Baek, A. J. Cho, S. Kim, G. Y. Lee, J. H. Han, T. M. Chung, S. H. Baek, C. Y. Kang, J. S. Kim, C. S. Hwang, S. K. Kim, *Chem. Mater.* **2020**, *32*, 9026.

[7] W. Wang, T. Zhang, A. Seliverstov, H. Zhang, Y. Wang, F. Wang, X. Peng, Q. Lu, C. Qin, X. Pan, Y. J. Zeng, C. Van Haesendonck, Z. Ye, *Adv. Electron. Mater.* **2020**, *6*, 1901020.

[8] V. Krishnamurthi, H. Khan, T. Ahmed, A. Zavabeti, S. A. Tawfik, S. K. Jain, M. J. S. Spencer, S. Balendhran, K. B. Crozier, Z. Li, L. Fu, M. Mohiuddin, M. X. Low, B. Shabbir, A. Boes, A. Mitchell, C. F. McConville, Y. Li, K. Kalantar Zadeh, N. Mahmood, S. Walia, *Adv. Mater.* **2020**, *32*, 2004247.

[9] K. C. Kwon, Y. Zhang, L. Wang, W. Yu, X. Wang, I.-H. Park, H. S. Choi, T. Ma, Z. Zhu, B. Tian, C. Su, K. P. Loh, *ACS Nano* **2020**, *14*, 7628.

[10] N. Higashitarumizu, H. Kawamoto, C. J. Lee, B. H. Lin, F. H. Chu, I. Yonemori, T. Nishimura, K. Wakabayashi, W. H. Chang, K. Nagashio, *Nat. Commun.* **2020**, *11*, 2428.

[11] H. Khan, N. Mahmood, A. Zavabeti, A. Elbourne, M. A. Rahman, B. Y. Zhang, V. Krishnamurthi, P. Atkin, M. B. Ghasemian, J. Yang, G. Zheng, A. R. Ravindran, S. Walia, L. Wang, S. P. Russo, T. Daeneke, Y. Li, K. Kalantar Zadeh, *Nat. Commun.* **2020**, *11*, 3449.

[12] A. S. Sarkar, A. Mushtaq, D. Kushavah, S. K. Pal, *npj 2D Mater. Appl.* **2020**, *4*, 1.

[13] J. R. Brent, D. J. Lewis, T. Lorenz, E. A. Lewis, N. Savjani, S. J. Haigh, G. Seifert, B. Derby, P. O'Brien, *J. Am. Chem. Soc.* **2015**, *137*, 12689.

[14] D. Lam, K. S. Chen, J. Kang, X. Liu, M. C. Hersam, *Chem. Mater.* **2018**, *30*, 2245.

[15] X. Fan, L. Su, F. Zhang, D. Huang, D. K. Sang, Y. Chen, Y. Li, F. Liu, J. Li, H. Zhang, H. Xie, *ACS Appl. Mater. Interfaces* **2019**, *11*, 47197.

[16] Y. Ye, Q. Guo, X. Liu, C. Liu, J. Wang, Y. Liu, J. Qiu, *Chem. Mater.* **2017**, *29*, 8361.

[17] D. Ma, J. Zhao, R. Wang, C. Xing, Z. Li, W. Huang, X. Jiang, Z. Guo, Z. Luo, Y. Li, J. Li, S. Luo, Y. Zhang, H. Zhang, *ACS Appl. Mater. Interfaces* **2019**, *11*, 4278.

[18] W. Huang, Z. Xie, T. Fan, J. Li, Y. Wang, L. Wu, D. Ma, Z. Li, Y. Ge, Z. N. Huang, X. Dai, Y. Xiang, J. Li, X. Zhu, H. Zhang, *J. Mater. Chem. C* **2018**, *6*, 9582.

[19] Z. Xie, D. Wang, T. Fan, C. Xing, Z. Li, W. Tao, L. Liu, S. Bao, D. Fan, H. Zhang, *J. Mater. Chem. B* **2018**, *6*, 4747.

[20] A. Jannat, F. Haque, K. Xu, C. Zhou, B. Y. Zhang, N. Syed, M. Mohiuddin, K. A. Messalea, X. Li, S. L. Gras, X. Wen, Z. Fei, E. Haque, S. Walia, T. Daeneke, A. Zavabeti, J. Z. Ou, *ACS Appl. Mater. Interfaces* **2019**, *11*, 42462.

[21] Y. Huang, L. Li, Y. H. Lin, C. W. Nan, *J. Phys. Chem. C* **2017**, *121*, 17530.

[22] Y. Ye, Y. Xian, J. Cai, K. Lu, Z. Liu, T. Shi, J. Du, Y. Leng, R. Wei, W. Wang, X. Liu, G. Bi, J. Qiu, *Adv. Opt. Mater.* **2019**, *7*, 1800579.
